# Skeletal and alveolar changes in conventional rapid palatal expansion (RPE) and miniscrew-assisted RPE (MARPE): a prospective randomized clinical trial using low-dose CBCT

**DOI:** 10.1186/s12903-022-02138-w

**Published:** 2022-04-08

**Authors:** Joo-Hee Chun, Amanda Cunha Regal de Castro, Sunmee Oh, Kyung-Ho Kim, Sung-Hwan Choi, Lincoln Issamu Nojima, Matilde da Cunha Gonçalves Nojima, Kee-Joon Lee

**Affiliations:** 1 Department of Orthodontics, Institute of Craniofacial Deformity, College of Dentistry, Yonsei University, 50-1 Yonsei-ro, Seodaemun-gu, Seoul, 03722 Korea; 2 Department of Pediatric Dentistry and Orthodontics, School of Dentistry, Universidade Federal Do Rio de Janeiro, Av. Pedro Calmon, 550, 21941-901 Rio de Janeiro, Brazil

**Keywords:** Palatal expansion technique, Orthodontic anchorage procedures, Cone-beam computed tomography, Cranial sutures, Alveolar bone loss

## Abstract

**Background:**

This prospective randomized clinical trial aimed to evaluate the immediate and short-term skeletal, dentoalveolar, and periodontal effects of rapid palatal expansion (RPE) and miniscrew-assisted RPE (MARPE) in adolescent and young adult patients.

**Methods:**

This study followed a two-arm, parallel, randomized clinical trial design that recruited patients with transverse maxillary deficiency in a 1:1 allocation ratio. Forty patients (14 men and 26 women) requiring maxillary expansion were randomly allocated to the RPE (n = 20, age = 14.0 ± 4.5) or MARPE (n = 20, age = 14.1 ± 4.2) groups. The assignment was performed via computer-generated block randomization, with a block size of four. Upon identical (35 turns) amount of expansion, low-dose cone-beam computed tomography images were taken before treatment (T0), immediately after expansion (T1), and after a 3-month consolidation period (T2). The primary outcome of this study comprised the assessment of midpalatal suture separation. Secondary outcomes included, skeletal, dentoalveolar, and periodontal measurements, which were performed at each time point.

**Results:**

The frequency of midpalatal suture separation was 90% (18/20) and 95% (19/20) for the RPE and MARPE groups, respectively. A greater increase in nasal width in the molar region (M-NW) and greater palatine foramen (GPF) was observed immediately after the expansion (T1-T0) and consolidation periods (T2-T0) in the MARPE group compared to the RPE group (*P* < 0.05). The MARPE and RPE groups showed similar dentoalveolar changes except for the maxillary width (PM-MW, M-MW). The MARPE group presented greater bilateral first premolar (PM-MW) and molar (M-MW) maxillary width in relation to the RPE group (*P* < 0.05). Through the expansion and consolidation periods (T2-T0), lesser buccal displacement of the anchor teeth was observed in the MARPE group (PM-BBPT, PM-PBPT, M-BBPT [mesial and distal roots], and M-PBPT)(* P* < 0.05).

**Conclusions:**

Midpalatal suture separation was observed in 90% and 95% of patients in the RPE and MARPE groups, respectively. Both RPE and MARPE groups exhibited significant triangular basal bone expansion and skeletal relapse during consolidation. Under identical amounts of expansion, the MARPE group showed lower decrease in the skeletal, dentoalveolar and periodontal variables after consolidation. The reinforcement of RPE with miniscrews contributes to the maintenance of the basal bone during consolidation period.

*Trial registration* WHO Institutional Clinical Trials Registry Platform (IRB No. KCT0006871 / Registration date 27/12/2021).

## Background

Orthodontic management of maxillary transverse deficiency was first reported in 1860 [1] based on the biomechanical principle of orthopedic separation of the two palatal halves by exerting expansion forces at the midpalatal and intermaxillary sutures [2, 3]. Since then, the rapid palatal expander (RPE) has been widely used and proven to be effective for the correction of maxillary posterior crossbites [4], transverse dental arch discrepancies [5], and deficient arch perimeter [6, 7].

In the conventional tooth-borne Hyrax RPE, anchorage is provided mainly by the maxillary first premolars and first molars, concentrating the expansion forces over the dentoalveolar area. Forces applied to these structures, therefore, are related to undesirable side effects, including dentoalveolar tipping; root resorption; periodontal side effects, such as reduction of alveolar bone height, bone dehiscence, and gingival recession [8, 9]; limited basal bone expansion effects; questionable long-term stability [10]; and tissue swelling and ulceration [11]. As overall maxillary expansion is a result of both skeletal and dentoalveolar displacement, the periodontal status after surgical and non-surgical treatments, especially in thin periodontal biotypes, is a major clinical concern [12–14]. Due to the known limitations, various bone-borne anchorage devices have been introduced and have shown clinical success [15–17]. Most of the currently available expanders are hybrid in nature and are composed of both miniscrews and tooth-borne parts. However, the role of miniscrews throughout the expansion and consolidation periods have not been well clarified, possibly because of the lack of well-controlled clinical trials.

Apart from the conventional notion that orthopedic midpalatal expansion in individuals over 15 years of age would be very challenging [18, 19], favorable sutural separation in postpubertal adolescents as well as in mature adults has been reported with success [15, 20]. This indicated that human facial sutures are likely to remain patent even in later decades of life, unlike the calvarial sutures that are largely obliterated around the twenties [21–23]. However, evidence demonstrating that the midpalatal suture changes from a wide and smooth suture to a progressively interdigitated pattern [18, 19] suggests that the same mechanical force may produce different biological and biomechanical effects in immature and mature bone, leading to potentially different side effects [24]. Therefore, the variation of appliance design according to age also needs to be studied.

Three-dimensional radiologic evaluation using currently available computed tomography provides valuable information; however, because of the high radiation dose, a controlled study using serial radiologic evaluation is largely unacceptable. Hence, this study followed a low-dose cone beam computed tomography (CBCT) protocol, which produces only a fraction of conventional CBCT radiation.

### Specific objectives and hypothesis

The aim of this prospective randomized clinical trial was to evaluate the immediate and short-term skeletal, dentoalveolar, and periodontal effects of RPE and miniscrew-assisted RPE (MARPE) in adolescent and young adult patients.

Considering the role of the miniscrews in maxillary expansion procedures, the alternative hypothesis of this study was that RPE and MARPE present different skeletal, dentoalveolar, and periodontal effects immediately after expansion and within a 3-month consolidation period.

## Methods

### Trial design

This study followed a two-arm, parallel, randomized clinical trial design that recruited patients with transverse maxillary deficiency in a 1:1 allocation ratio. The clinical trial was registered as number KCT0006871 at the WHO International Clinical Trial Registry Platform. This study was conducted according to the Consolidated Standards of Reporting Trials (CONSORT) guidelines [25], in full compliance with the Declaration of Helsinki and was approved by the Institutional Review Board of Yonsei Dental Hospital (IRB No. 2-2016-0045).

### Participants

The patients were recruited in the department of Orthodontics, Yonsei Dental Hospital, Seoul, Korea, from February of 2017 to February of 2018. To be eligible, the patients had to meet the following inclusion criteria: (1) a maxillo-mandibular transverse discrepancy indicated by buccal edge-to-edge bite or crossbite, (2) patients aged 7 to 25 years, (3) good oral hygiene and healthy periodontal tissues, (4) no prior history of orthodontic treatment and/or orthognathic surgery, (5) no significant dentofacial anomalies, such as a cleft lip or palate, and (6) patients who consented to participate in this study. The exclusion criteria were as follows: (1) those under the age of 7 years or over 25 years, (2) those who do not have fixed anchor teeth, (3) periodontal disease, (4) previous orthodontic treatment, (5) maxillofacial deformity, and (6) those who did not consent to participate in this study.

### Interventions

**Fig. 1:**
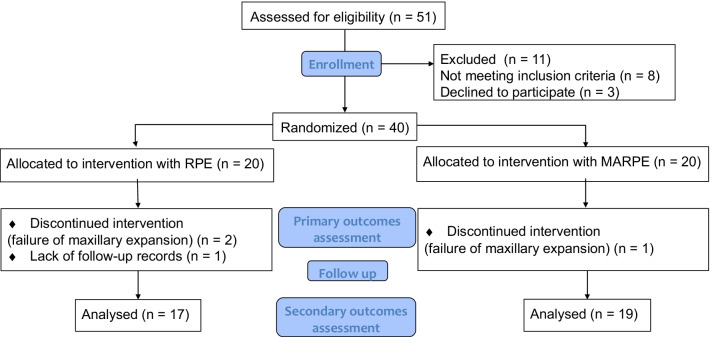
CONSORT flow diagram

**Fig. 2:**
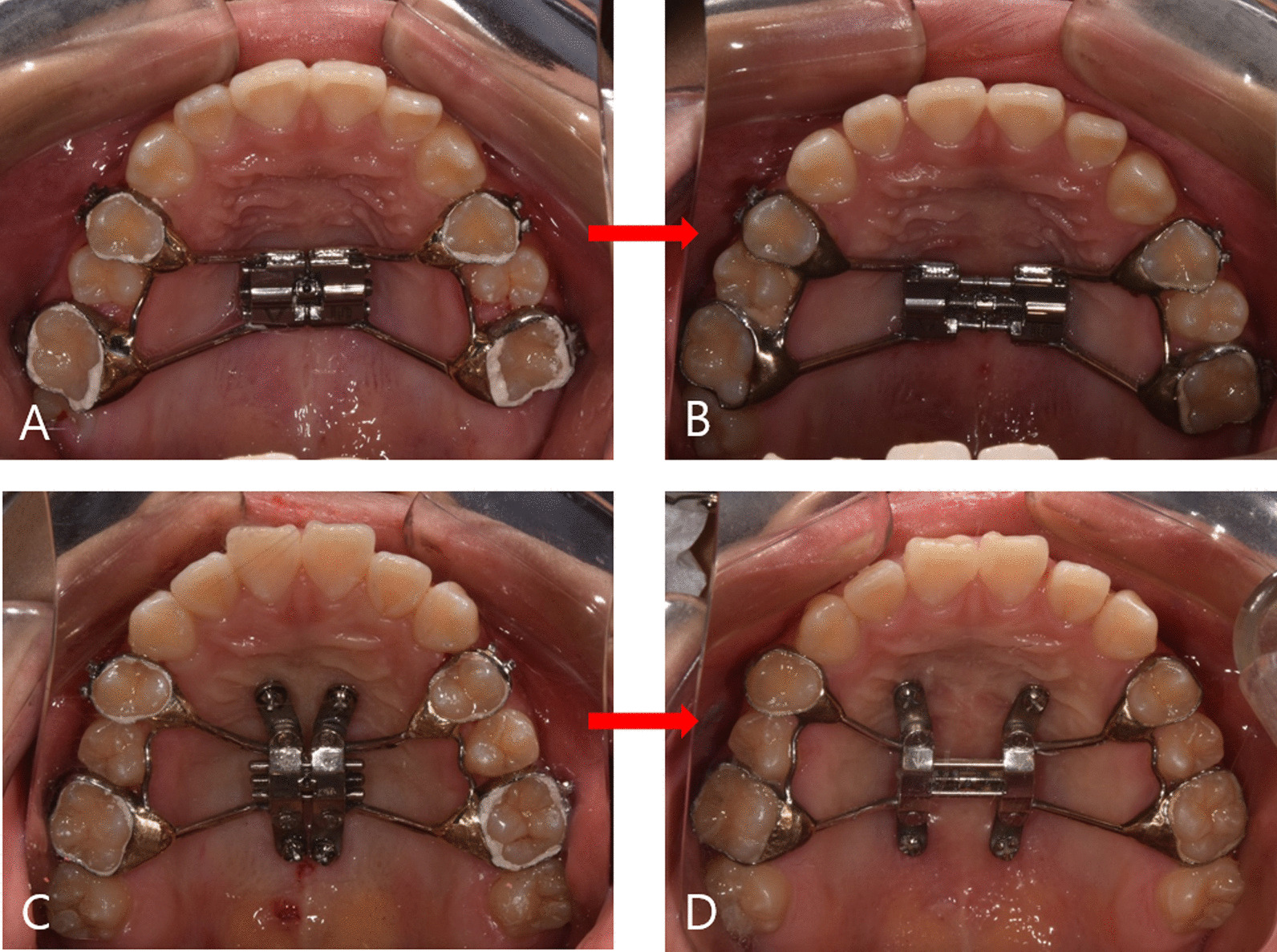
**a**, **b**, tooth-borne rapid palatal expansion (RPE) before and after expansion, respectively. **c**, **d**, miniscrew-assisted rapid palatal expansion (MARPE) before and after expansion, respectively

The tooth-borne RPE device consisted of four bands placed one each on the maxillary first premolars and first permanent molars. Regular RPE was fabricated using Hyrax expander (Dentaurum, Ispringen, Germany) (Fig. 1, 2a and b). The MARPE device had four rigid plates with screw holes extending from the jackscrew body to accommodate four bone screws as well as four rigid arms soldered on the maxillary first premolars and first molars (Biomaterials Korea, Seoul, Korea). Following MARPE cementation, four self-drilling bone screws (1.8 mm diameter and 9 mm length for anterior region, 7 mm length for posterior region, respectively) were inserted perpendicularly to the center of the screw hole under local infiltration anesthesia (Fig. 2c and d). The anterior miniscrews were placed medially to the first premolars, on a line parallel to the midpalatal suture and passing between the central and lateral incisors, the posterior miniscrews were placed just lateral to the midpalatal suture in the first molar region.

The RPE and MARPE devices were activated by one-quarter of a turn (0.20 mm/turn) once a day. Both RPE and MARPE were activated 35 times, which corresponded to 7.0 mm of hyrax screw expansion. After active expansion, the devices were maintained for a 3-month consolidation period to enable connective tissue remodeling of the suture. The devices were activated by the same orthodontist (J-H. C.) in both groups.

### Outcomes

The primary outcome of this study involved midpalatal suture opening. During the activation period, patients were observed weekly in order to confirm midpalatal suture opening. Failure of maxillary expansion using RPE or MARPE was defined when radiographic signs of midpalatal suture opening were not observed in the periapical view at 4 weeks from the first activation. In the event of the failure of midpalatal suture opening using RPE or MARPE devices, expansion was discontinued and the orthodontic treatment plan was revised so that the treatment could be safely completed. The secondary outcomes comprised skeletal, dentoalveolar, and periodontal evaluations of CBCT images.

### CBCT protocol and analysis

CBCT images (Alphard 3030, Asahi Roentgen Ind. Co., Ltd., Kyoto, Japan) were recorded by following a low-dose protocol (exposure time: 17 s, 3.0 mA, 80 kV, field of view [FOV]: 200 × 200 mm^2^, voxel size: 0.39 mm) before treatment (T0), immediately after expansion (T1), and after a 3-month consolidation period (T2) to ensure that the total radiation dose of repeated CBCT imaging during the experiment did not exceed the recommended annual dose limit (1 mSv) [26, 27].

**Fig. 3:**
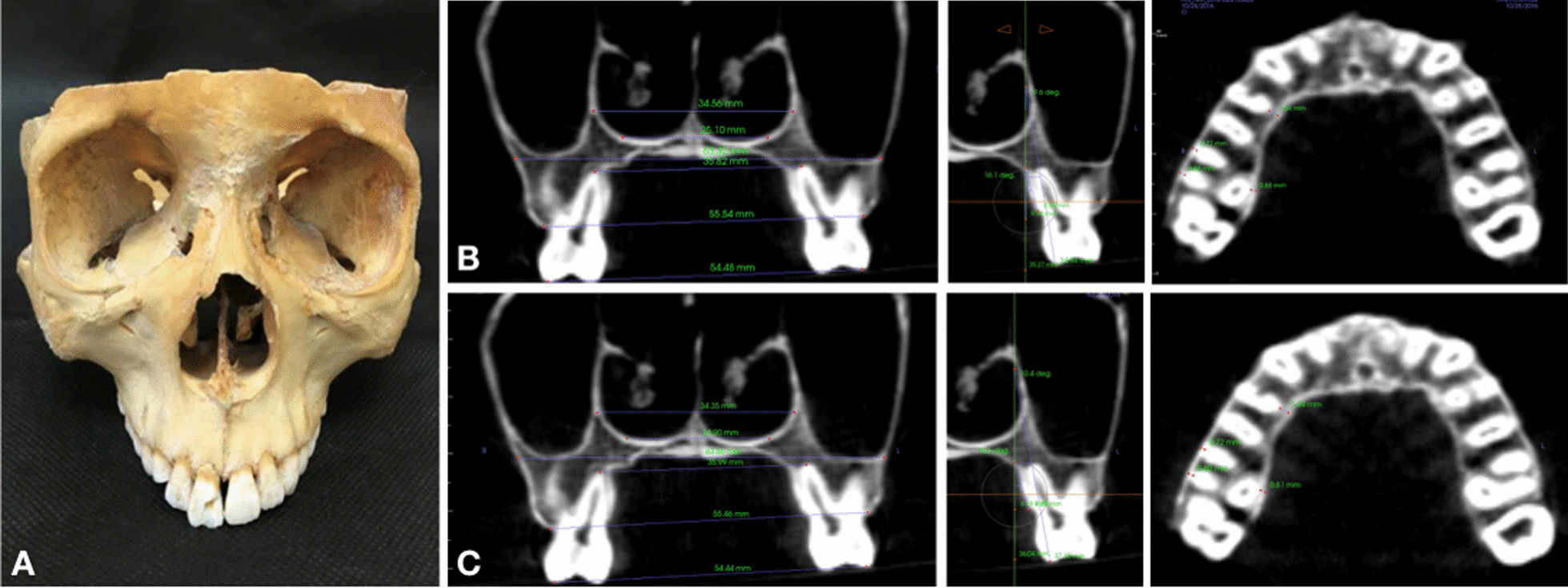
Preliminary study. **a** Human dry skull; **b** CBCT images in standard mode; **c** CBCT images in low-dose mode

A preliminary study was conducted to determine the accuracy of the low-dose CBCT [28]. CBCT images of a human dry skull were recorded with the low-dose and standard-dose modes (exposure 17 s, 10.0 mA, 80 kV, FOV 200 × 200 mm^2^, voxel size 0.39 mm). A single examiner performed 11 measurements in both sets of images to determine the agreement rate between low-dose and standard-dose protocols (Fig. 3). The intraclass correlation coefficient (ICC) was greater than 0.99 in all the pilot study measurements.

Digital Imaging and Communications in Medicine (DICOM) file image reconstruction (slice thickness 0.5 mm) and analysis were performed with InVivo5® (Anatomage, San Jose, CA, USA) and Dolphin Imaging® (version 11.95 Premium, Chatsworth, CA, USA) software.

**Fig. 4:**
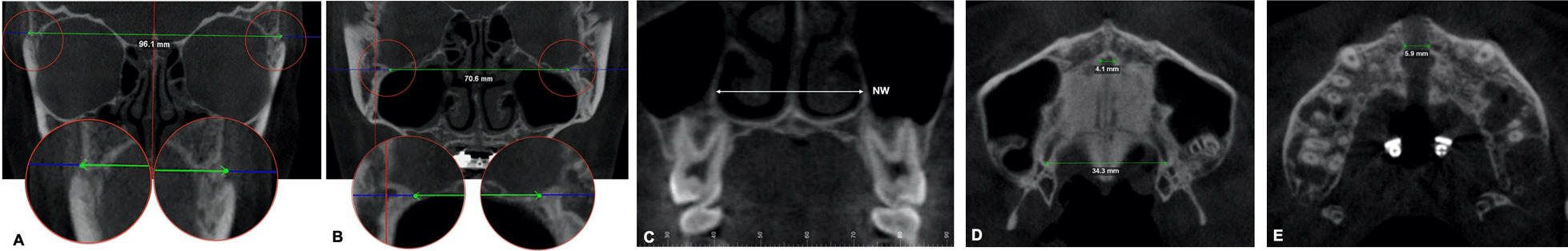
CBCT images illustrating skeletal variables: **a** Frontozygomatic suture. **b** Zygomaticomaxillary suture. **c** Nasal width. **d** Nasopalatine and greater palatine foramen. **e** Mid palatal suture gap

**Fig. 5:**
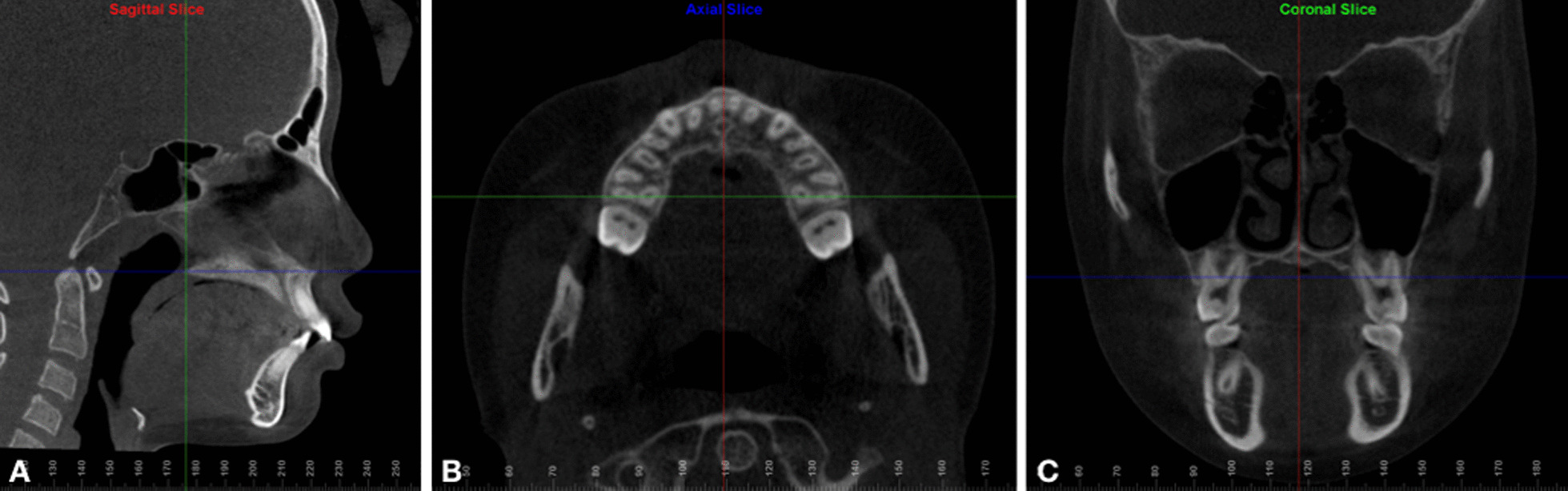
Re-orientation of CBCT images. Reoriented as parallel to the palatal plane (**a** sagittal section), passing through the root apices of both maxillary first molars (**b** axial section), and parallel to the hard palate (**c** coronal section)

**Fig. 6:**
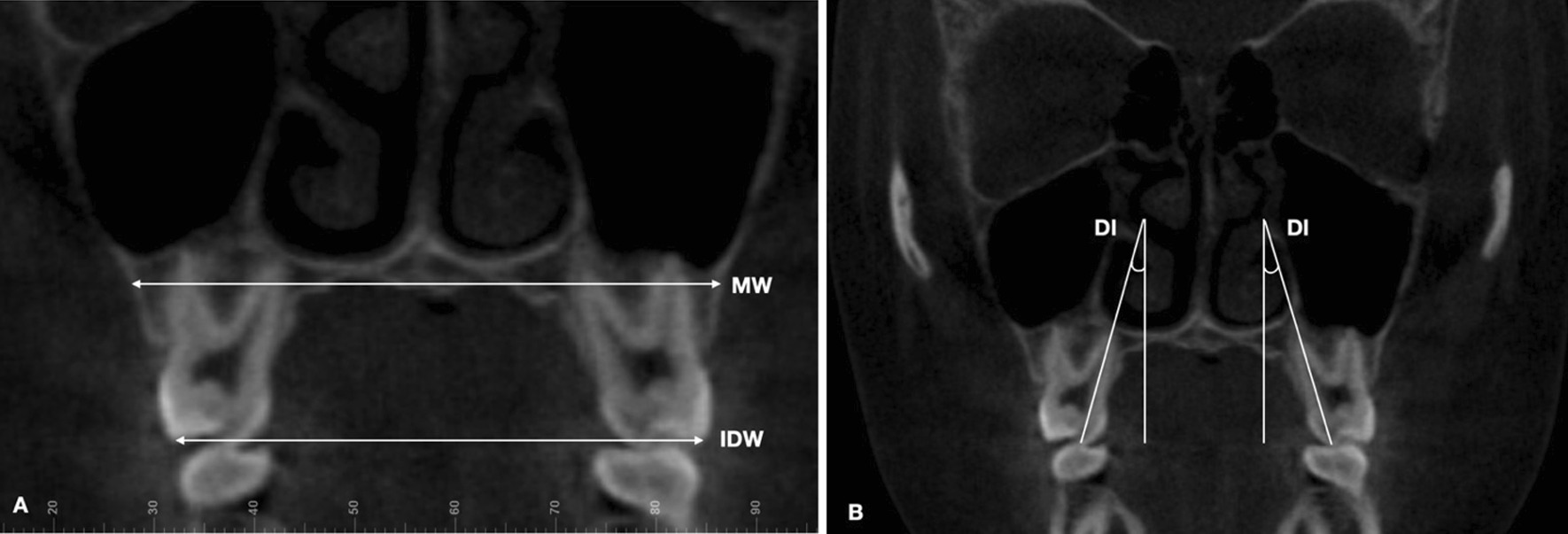
CBCT images illustrating dentoalveolar variables: **a** MW, maxillary width at hard palate; IDW, interdental width; **b** DI, dental inclination

**Fig. 7:**
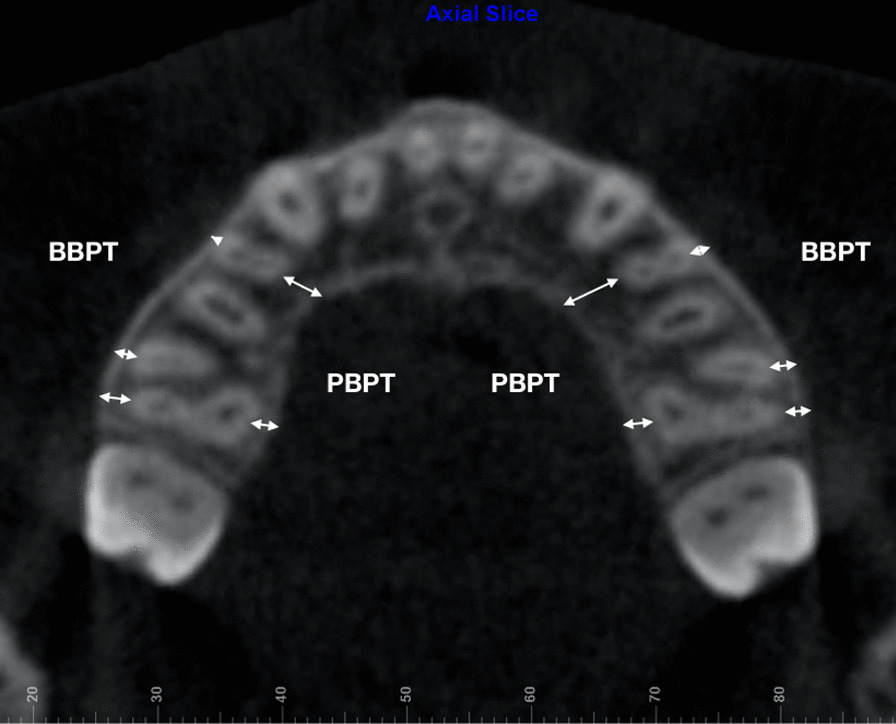
CBCT image illustrating periodontal variables: BBPT, buccal bone plate thickness; and PBPT, palatal bone plate thickness

Skeletal (frontozygomatic suture–FZS, zygomaticomaxillary suture–ZMS, nasal width–NW, nasopalatine foramen–NPF, greater palatine foramen–GPF, and midpalatal suture–MPS), dentoalveolar (maxillary width–MW, interdental width–IDW, and dental inclination–DI), and periodontal measurements (buccal bone plate thickness – BBPT; and palatal bone plate thickness – PBPT), described in Table 1, were performed by experienced operators (J-H. C. and A.C.R.C.). A protocol of image orientation and analysis (Figs. 4, 5, 6, 7) was developed for FZS, ZMS, NPF, GPF, and MPS, whereas NW, dentoalveolar, and periodontal parameters were analyzed according to previously published methods [12, 13, 17, 29] as detailed in Table 1.

**Table 1:** Definitions of the parameters measured in the study

Measurement	Definition
**Skeletal measurements**	
FZS (Frontozygomatic suture)	Distance (mm) between the two points located at the edges of the zygomatic process of the frontal bone measured in the coronal section
ZMS (Zygomaticomaxillary suture)	Distance (mm) between the two points located at the superior edges of ZMS measured in the coronal section
NW (Nasal width)	Distance (mm) at the widest portion of the nasal aperture parallel to the hard palate
	PM-NW and M-NW were measured in both upper first premolars (PM) and first molars (M) in the coronal section, respectively
NPF (Nasopalatine foramen)	Distance (mm) between the points located at the greater diameter of the nasopalatine foramen, bilaterally, measured in the axial section
GPF (Greater palatine foramen)	Distance (mm) between the points located at the posterior cortical of the greater palatine foramen, bilaterally, measured in the axial section
MPS ( Midpalatal suture gap)	Distance (mm) of midpalatal suture gap at upper central incisors apical level, measured in the axial section, immediately after expansion (T1)
**Dentoalveolar measurements**	
MW(Maxillary width)	Distance (mm) of maxillary width tangent to the hard palate
	PM-MW and M-MW were measured on both upper first premolars (PM) and first molars (M) in the coronal section
IDW (Interdental width)	Distance (mm) between the right and left buccal cusp tips
	PM-IDW and M-IDW were measured on both upper first premolars (PM) and first molars (M) in the coronal section
DI (Dental inclination)	Angle (°) between the line passing through the palatal cusp tip and palatal root apex, and the vertical line perpendicular to the hard palate measured on upper first molars in the coronal section
**Periodontal measurements**	
BBPT(Buccal bone plate thickness)	The shortest distance (mm) between the buccal cortical plate and the buccal root surface
	PM-BBPT, M-BBPT (Mesial and Distal root) were measured on upper first premolars and mesial and distal first molars roots
PBPT(Palatal bone plate thickness)	The shortest distance (mm) between the palatal cortical plate and the palatal root surface
	PM-PBPT and M-PBPT were measured on upper first premolars and palatine first molars roots

### Follow up

All patients were followed up for secondary outcomes (T1 and T2), so that the difference between the time points indicated the effects of RPE and MARPE devices over the time of the study. After the activation of the expansion device was completed, data collection was performed for a follow-up period of 3 months to evaluate possible side effects. After the removal of the expansion device, the planned orthodontic treatment proceeded with braces and was subsequently followed up for about 18 months until the treatment was completed.

### Sample size

Based on a previous study by Lin et al. [16], a minimum sample size of 16 patients was required (G*Power 3.1.9.6, Dusseldorf, Germany), considering an alpha of 0.05, a power of 80%, and an effect size of 0.94 to detect differences in midpalatal suture separation at the hard palatal level with bone-borne and tooth-borne appliances using a Mann–Whitney U-test.

### Randomization

The assignment to RPE and MARPE groups was performed via a computer-generated block randomization procedure, with a block size of four. Allocation concealment comprised opaque, sealed, and sequenced numbered envelopes [30].

### Blinding

As the operator, patients and outcome examiners were aware of the type of maxillary expansion device, blinding could not be performed.

### Statistical analysis

All statistical analyses were performed with IBM SPSS software for Windows (version 20.0; SPSS Inc., Chicago, Illinois, USA). Descriptive statistics were used to describe each variable analyzed in the study. The Shapiro–Wilk test was used to verify the normality of data distribution. Mann–Whitney U tests and Pearson chi-square tests were applied to baseline characteristics. The primary outcome was expressed as frequencies and evaluated with the Pearson chi-square test. The repeated-measures analysis of variance (RMANOVA) with Bonferroni correction (α = 0.016) and Friedman’s analysis of variance were used to detect changes in secondary outcomes: skeletal, dentoalveolar, and periodontal measurements over the study time points (T0, T1, and T2). Independent *t*-tests and Mann–Whitney U tests were performed for intergroup comparisons (RPE/MARPE). The level of statistical significance was set at *P* ≤ 0.05. Pearson’s correlation test was used to evaluate the relationship between the MPS gap at T1 and the mean difference in periodontal variables between T1-T0 and T2-T0 (α = 0.05). All measurements were repeated in 30% of the samples after a 2-week interval. The ICC was calculated and was greater than 0.80 for all the study variables.

## Results

### Participant flow

Of a total of 51 adolescent and young adult patients who were screened, eight patients did not meet the inclusion criteria and three declined to participate. Thus, forty patients were enrolled in the study. Among the forty patients, twenty patients each were allocated to the RPE and MARPE groups. Three patients (two from the RPE group and one from the MARPE group) were excluded from the secondary outcome evaluation due to the failure of the midpalatal suture opening. Therefore, expansion was discontinued and the orthodontic treatment plan was revised. Additionally, another patient from the RPE group was excluded from the secondary outcomes evaluation due to missing the follow-up records (Fig. 1).

### Baseline data

The mean age of the study participants was 14.0 ± 4.3 years. The RPE group consisted of 20 patients (6 men, 14 women) with a mean age of 14.0 ± 4.5 years. The MARPE group consisted of 20 patients (8 men, 12 women) with a mean age of 14.1 ± 4.2 years. There was no statistically significant difference between age and sex distribution across the groups (Table 2).

**Table 2:** Characteristics of subjects

	Total (N = 40)		
	RPE (N = 20)	MARPE (N = 20)	P
Age (y)	14.0 ± 4.5	14.1 ± 4.2	0.968*
Sex			
Men (n (%))	6 (30.0)	8 (40.0)	0.507^†^
Women (n (%))	14 (70.0)	12 (60.0)	

RPE, Rapid palatal expansion; MARPE, mini-screw assisted rapid palatal expansion. Values are mean ± Standard deviation or n (%).*P-value for the Mann–Whitney U test. ^†^P-value for the Pearson chi-square test

### Primary outcomes

In the RPE group, midpalatal suture separation occurred in 18 out of 20 patients. The MARPE group demonstrated a successful midpalatal suture opening in 19 out of 20 patients. The frequency of midpalatal suture separation was 90% and 95% for RPE and MARPE, respectively, without statistical differences between the groups (*P* > 0.99).

### Skeletal changes

**Fig. 8:**
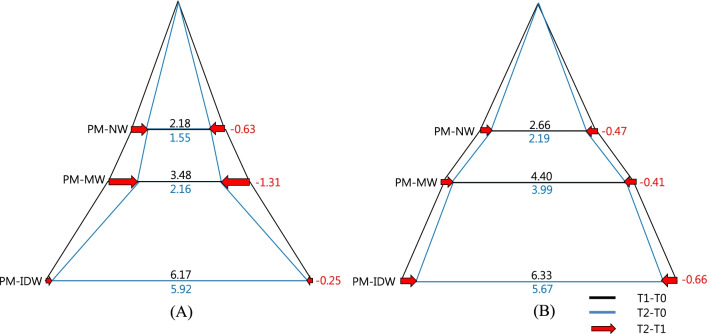
Diagram of transverse change of the maxillary first premolars at each timepoint in the coronal section. Values are the mean differences (mm). **a** RPE, **b** MARPE; RPE, rapid palatal expansion; MARPE, mini-screw assisted rapid palatal expansion; T0, before treatment; T1, immediately after expansion; T2, after a 3-month consolidation period

**Fig. 9:**
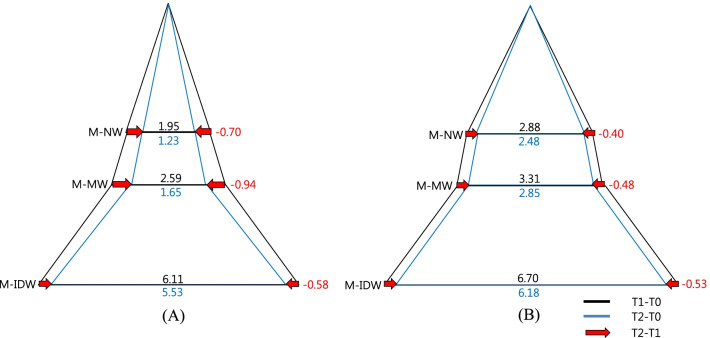
Diagram of transverse change of the maxillary first molars at each timepoint in the coronal section. Values are the mean differences (mm). **a** RPE, **b** MARPE; RPE, rapid palatal expansion; MARPE, mini-screw Assisted rapid palatal expansion; T0, before treatment; T1, immediately after expansion; T2, after a 3-month consolidation period

Immediately after expansion (T1-T0), the nasal width in relation to the bilateral first molar region (M-NW) was found to be significantly increased in the MARPE group compared to the RPE group (*P* < 0.05). Significant basal bone expansion was noted at the ZMS, NW in relation to the bilateral premolar region (PM-NW), M-NW, NPF, and GPF in both groups (*P* = 0.016), but not at the FZS, implying an overall triangular maxillary expansion. The MARPE group presented a significant increase at the GPF compared to that in the RPE group (*P* < 0.05), (Table 3, Figs. 8, 9). Following the 3-month consolidation period (T2-T1), the RPE and MARPE groups presented reductions in PM-NW, M-NW, and NPF over time (*P* < 0.05) (Table 3, Figs. 8, 9).

**Table 3:** Descriptive statistics of skeletal variables according to intervention groups at different timepoints

Variables	Timepoint	RPE (N = 17)	MARPE (N = 19)	*P *value
FZS (mm)	T1-T0	0.36 ± 0.52	0.05 ± 0.38	0.076
	T2-T1	− 0.21 ± 0.62	0.10 ± 0.48	0.100
	T2-T0	0.14 ± 0.52	0.15 ± 0.58	0.962
ZMS (mm)	T1-T0	1.04 ± 1.01^†^	1.49 ± 0.83^†^	0.157
	T2-T1	− 0.14 ± 0.80	− 0.07 ± 0.61	0.697
	T2-T0	0.89 ± 0.83^†^	1.41 ± 0.87^†^	0.080
PM-NW (mm)	T1-T0	2.18 ± 0.99^†^	2.66 ± 0.63^†^	0.098
	T2-T1	− 0.63 ± 0.43^††^	− 0.47 ± 0.40^††^	0.285
	T2-T0	1.55 ± 1.02^†^	2.19 ± 0.84^†^	0.045*
M-NW (mm)	T1-T0	1.95 ± 0.81^†^	2.88 ± 0.82^†^	0.002*
	T2-T1	− 0.70 ± 1.26^††^	− 0.40 ± 0.34 ^††^	0.827
	T2-T0	1.23 ± 1.66	2.48 ± 0.84^††^	0.003**
NPF (mm)	T1-T0	2.85 ± 1.95^†^	3.22 ± 1.20^†^	0.495
	T2-T1	− 1.42 ± 1.28^††^	− 1.15 ± 1.42^††^	0.346
	T2-T0	1.43 ± 1.50^†^	2.07 ± 1.37^†^	0.199
GPF(mm)	T1-T0	1.84 ± 1.31^†^	2.70 ± 1.18^†^	0.048*
	T2-T1	0.31 ± 1.05	− 0.30 ± 0.75	0.639
	T2-T0	1.52 ± 0.85^†^	2.40 ± 0.96^†^	0.008*

Descriptive statistics represented as mean ± standard deviation

^†^Indicate statistical significance with repeated measures analysis of variance with Bonferroni correction (α = 0.016)

^††^ Indicate statistical significance with Friedman’s analysis of variance by ranks (α = 0.05)

*Indicate statistical significance with independent t-test at each time point (*P* < 0.05)

**Indicate statistical significance with Mann–Whitney U test at each time point (*P* < 0.05)

Abbreviations: RPE, rapid palatal expansion; MARPE, mini-screw assisted rapid palatal expansion; T0, before treatment; T1, immediately after expansion; T2, after a 3-month consolidation period

Overall (T2-T0), throughout the expansion and consolidation periods, both the treatment groups showed significant increases in all dimensions (*P* < 0.05), except at the FZS and in the M-NW in the RPE group. Significant intergroup differences were observed in the PM-NW, M-NW, and GPF with significantly greater increases in these parameters in the MARPE group compared to the RPE group (*P* < 0.05) (Table 3, Figs. 8, 9).

### Dentoalveolar changes

As a result of successful orthopedic expansion, a significant increase was observed in the maxillary width in the bilateral first premolar (PM-MW) and molar region (M-MW) (P<0.05). Both the RPE and MARPE groups presented significant increases of M-MW immediately after expansion (T1-T0), with particularly greater values observed in the MARPE group. Both groups presented significantly greater transverse dental dimensions in the premolar (PM-IDW) and molar (M-IDW) areas immediately after expansion (T1-T0) (P<0.05). The amount of expansion ranged from 6.1 to 6.3 mm in the premolar region (PM-IDW) and from 5.9 to 6.7 mm in the molar region (M-IDW). Moreover, this dimension is slightly reduced during the consolidation period (T2-T1) (Table 4, Figs. 8, 9).

**Table 4:** Descriptive statistics of dentoalveolar variables according to intervention groups at different timepoints

Variables	Timepoint	RPE (N = 17)	MARPE (N = 19)	*P *value
PM-MW (mm)	T1-T0	3.48 ± 1.77^††^	4.40 ± 2.25^††^	0.175
	T2-T1	− 1.31 ± 1.01^††^	− 0.41 ± 0.51	0.002**
	T2-T0	2.16 ± 2.00	3.99 ± 2.46^††^	0.019**
PM-IDW (mm)	T1-T0	6.17 ± 1.52^††^	6.33 ± 1.41^††^	0.594
	T2-T1	− 0.25 ± 0.50	− 0.66 ± 0.64^††^	0.071
	T2-T0	5.92 ± 1.47^†^	5.67 ± 1.43^†^	0.602
M-MW (mm)	T1-T0	2.59 ± 1.17^†^	3.31 ± 0.82^†^	0.038*
	T2-T1	− 0.94 ± 0.61^†^	− 0.48 ± 0.70	0.043*
	T2-T0	1.65 ± 1.34^†^	2.85 ± 0.98^†^	0.004*
M-IDW (mm)	T1-T0	6.11 ± 1.29^†^	6.70 ± 1.10^†^	0.150
	T2-T1	− 0.58 ± 0.55	− 0.53 ± 0.49	0.851
	T2-T0	5.53 ± 1.47^†^	6.18 ± 1.23^†^	0.159
M-DI (°)	T1-T0	3.40 ± 2.47^††^	3.94 ± 1.61^††^	0.219
	T2-T1	− 0.98 ± 1.25	− 1.92 ± 1.61^††^	0.208
	T2-T0	2.41 ± 2.55^††^	2.02 ± 1.11^††^	0.975

Descriptive statistics represented as mean ± standard deviation

^†^Indicate statistical significance with repeated measures analysis of variance with Bonferroni correction (α = 0.016)

^††^ Indicate statistical significance with Friedman’s analysis of variance by ranks (α = 0.05)

*Indicate statistical significance with independent t-test at each time point (*P* < 0.05)

**Indicate statistical significance with Mann–Whitney U test at each time point (*P* < 0.05)

Abbreviations: RPE, rapid palatal expansion; MARPE, mini-screw assisted rapid palatal expansion; T0, before treatment; T1, immediately after expansion; T2, after a 3-month consolidation period

During consolidation (T2-T1), the MARPE group presented a lower decrease in the PM-MW and M-MW compared to that in the RPE group (*P* < 0.05), suggesting a greater alveolar relapse in the RPE group.

Hence, through the expansion and consolidation periods (T2-T0) there were no significant intergroup (RPE vs MARPE) differences in all the dental dimensions except for the changes in the PM-MW and M-MW (*P* < 0.05) (Table 4, Figs. 8, 9).

The PM-IDW, M-IDW and upper first molar axes (M-DI) did not present statistical significance between RPE and MARPE groups. The M-DI increased immediately after expansion (T1-T0) followed by a minor decrease during the consolidation period (T2-T1) in both groups, which resulted in a similar overall M-DI (Table 4, Figs. 8, 9).

### Periodontal changes

In general, the premolar BBPT (PM-BBPT) and molar BBPT (M-BBPT) on mesial and distal roots reduced throughout the expansion and consolidation periods (T2-T0) regardless of expander types, possibly because of a skeletal relapse tendency.

**Fig. 10:**
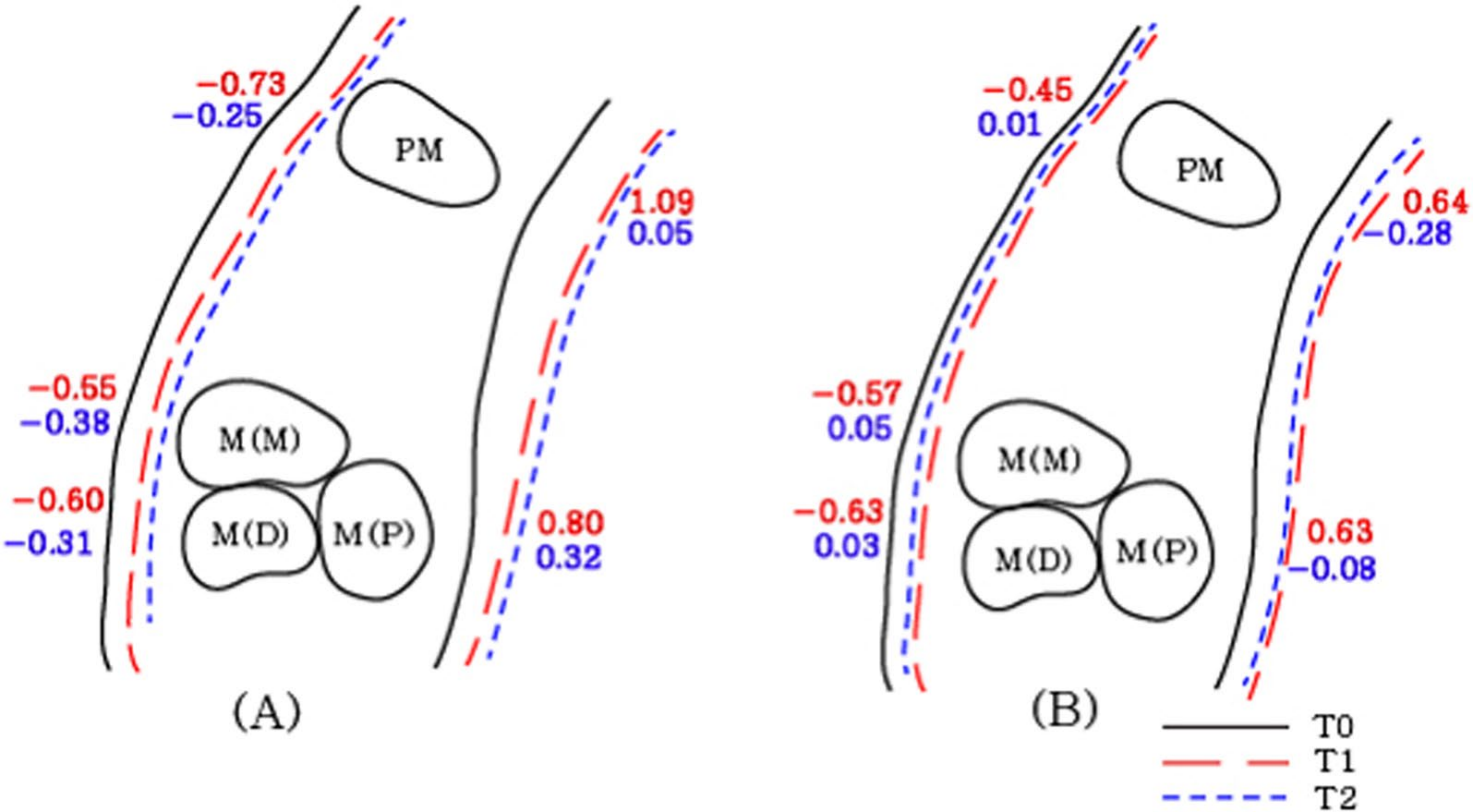
Diagram of periodontal changes of the anchor teeth at each timepoint in the axial section. Values are the mean differences (mm). **a** RPE. **b** MARPE; RPE, rapid palatal expansion; MARPE, mini-screw assisted rapid palatal expansion; T0, before treatment; T1, immediately after expansion; T2, after a 3-month consolidation period

Specifically, during expansion (T1-T0), all BBPT values are reduced by 0.6 mm on average indicating buccal displacement of the anchor premolars and molars within the alveolar bone. Conversely, all PBPT values increased in both expander types. A significant intergroup difference was observed only in the premolar area (PM-BBPT, *P* < 0.05) (Table 5, Figs. 10).

**Table 5:** Descriptive statistics of periodontal variables according to intervention groups at different timepoints

Variables	Timepoint	RPE (N = 17)	MARPE (N = 19)	*P *value
PM-BBPT (mm)	T1-T0	− 0.73 ± 0.36^†^	− 0.45 ± 0.30^†^	0.016*
	T2-T1	− 0.25 ± 0.47	0.01 ± 0.39	0.076
	T2-T0	− 0.96 ± 0.44^†^	− 0.43 ± 0.38^†^	< 0.001*
PM-PBPT (mm)	T1-T0	1.09 ± 0.89^††^	0.64 ± 0.56^††^	0.087
	T2-T1	0.05 ± 0.52	− 0.28 ± 0.58	0.083
	T2-T0	1.16 ± 0.62^††^	0.35 ± 0.43^††^	< 0.001**
M-BBPT (Mesial root) (mm)	T1-T0	-0.55 ± 0.39^††^	− 0.57 ± 0.39^††^	0.707
	T2-T1	− 0.38 ± 0.30^††^	0.05 ± 0.32	< 0.001**
	T2-T0	− 0.91 ± 0.40^†^	− 0.45 ± 0.56^†^	0.007*
M-BBPT (Distal root) (mm)	T1-T0	− 0.60 ± 0.34^†^	− 0.63 ± 0.36^†^	0.824
	T2-T1	− 0.31 ± 0.35	0.03 ± 0.29	0.005**
	T2-T0	− 0.90 ± 039^†^	− 0.54 ± 0.57^†^	0.032*
M-PBPT (mm)	T1-T0	0.80 ± 0.61^††^	0.63 ± 0.32^††^	0.661
	T2-T1	0.32 ± 0.32	− 0.08 ± 0.38	0.001**
	T2-T0	1.10 ± 0.57^†^	0.55 ± 0.30^†^	0.001*

Descriptive statistics represented as mean ± standard deviation

^†^Indicate statistical significance with repeated measures analysis of variance with Bonferroni correction (α = 0.016)

^††^ Indicate statistical significance with Friedman’s analysis of variance by ranks (α = 0.05)

*Indicate statistical significance with independent t-test at each time point (*P* < 0.05)

**Indicate statistical significance with Mann–Whitney U test at each time point (*P* < 0.05)

Abbreviations: RPE, rapid palatal expansion; MARPE, mini-screw assisted rapid palatal expansion; T0, before treatment; T1, immediately after expansion; T2, after a 3-month consolidation period

During consolidation (T2-T1), the bone thickness changes were distinguished according to the expander types. Both M-BBPT in relation to the mesial and distal roots reduced in the RPE group, which was contrasted by a slight decrease or increase in the MARPE group, resulting in significant intergroup difference (*P* < 0.05). A similar pattern was observed in the premolar area with no statistical significance. This result indicates that consolidation with the use of the MARPE device may lead to less buccal alveolar bone loss (Table 5, Figs. 10).

Through the expansion and consolidation periods (T2-T0), the difference between the expander types was remarkable, exhibiting statistical significance in all measurements (PM-BBPT, PM-PBPT, M-BBPT [mesial and distal roots], and M-PBPT) (*P* < 0.05). This implied that lesser buccal displacement of the anchor teeth occurs within the alveolar bone in the MARPE group for a given amount of expansion.

To examine the possible relationship between the amount of midpalatal expansion and the changes in the alveolar bone plate, Pearson’s correlation analysis was conducted. Interestingly, a significant positive correlation was found only in the MARPE groups during expansion (T1-T0) (PM-PBPT in MARPE group). In contrast, a lack of correlation was observed in the RPE group (Table 6).

**Table 6:** Pearson correlations between periodontal variables and midpalatal suture gap according to intervention groups

Periodontal variables			MPS (T1)	
			RPE (N = 17)	MARPE (N = 19)
PM-BBPT (mm)	T1-T0	r	0.059	− 0.040
		Sig	0.823	0.872
	T2-T0	r	0.225	− 0.016
		Sig	0.384	0.949
PM-PBPT (mm)	T1-T0	r	0.403	0.503
		Sig	0.109	0.028*
	T2-T0	r	0.384	0.298
		Sig	0.128	0.215
M-BBPT (Mesial root) (mm)	T1-T0	r	− 0.073	− 0.341
		Sig	0.779	0.153
	T2-T0	r	0.009	− 0.190
		Sig	0.972	0.435
M-BBPT (Distal root) (mm)	T1-T0	r	0.107	− 0.407
		Sig	0.684	0.084
	T2-T0	r	0.029	− 0.287
		Sig	0.912	0.234
M-PBPT (mm)	T1-T0	r	0.069	0.286
		Sig	0.792	0.236
	T2-T0	r	− 0.058	0.444
		Sig	0.826	0.057

*Indicate statistical significance with Pearson’s correlation test (*P < 0.05). Abbreviations: MPS (T1), midpalatal suture gap at T1; RPE, rapid palatal expansion; MARPE, mini-screw assisted rapid palatal expansion; T0, before treatment; T1, immediately after expansion; T2, after a 3-month consolidation period; r, Pearson’s correlation; Sig, significance

## Discussion

One of the purposes of orthopedic maxillary expansion is to secure buccolingual alveolar surroundings along with lateral displacement of the buccal segment for the establishment of proper transverse dimension in the maxilla. In a previous study, tooth-borne expanders have shown unavoidable side effects, such as bony dehiscences and short-term skeletal relapse at the end of the consolidation period in children due to anchor teeth’s buccal displacement [12], which can lead to a reduction in the BBPT. This study implicated that the side effects on the anchor teeth may persist even in successfully separated sutures throughout the expansion and consolidation phase.

On the other hand, purely bone-borne expanders, despite the absence of the side effects in the alveolar bone, exhibit significantly less increase in the intermolar width compared to the conventional tooth-borne expanders in adolescents [31]. Accordingly, a tooth-and-bone-borne maxillary expander, which is a simple combination of a conventional expander and bone-borne anchorage devices, has shown favorable orthopedic and dentoalveolar expansion even in young adults [20, 32]. Nonetheless, the respective roles of the tooth-borne anchorage parts and miniscrews have been unclear. In order to analyze the changes during expansion and consolidation, a comprehensive three-dimensional observation of the maxilla at the end of the expansion and consolidation periods was necessary; therefore, a low-dose CBCT was crucial to evaluate the changes at all three time points.

When compared to adults, children and adolescents are highly sensitive to radiation during their growing stages and therefore they may be affected by the conventional radiation dose. Hence, in this study, an effort was made to reduce the patients’ radiation exposure by obtaining CBCT images with a low-dose protocol. In 2008, Palomo et al. [33] reported that, when the electric current was lowered to 2 mA, the average effective dose delivered to each organ could be lowered about 0.18 times compared to a 15 mA current. In the same year, Ballanti et al. [26] reported their study, which they performed with a low-dose CT protocol for dental care, 80 kV compared with 120 kV resulted in reduced total radiation without compromising the information provided by the diagnostic images. Additionally, a preliminary study conducted with human skulls confirmed that difference observed between low-dose and conventional CBCT images with a voxel size of 0.39 mm was minor and did not significantly affect the measurements of periodontal tissue and dental units [28].

Suture separation was observed in 92.5% of the participants (37/40), which was similar to the results of previous studies [20, 34]. The frequency of midpalatal suture separation was 90% and 95% for the RPE and MARPE groups, respectively.

In the present study, the initial expansion pattern was slightly triangular in the coronal plane as well as in the axial plane. In the coronal plane, expansion of the overall craniofacial structure may be depicted as a triangular pattern with the base at the level of the dental arch. The increase in the nasal width, as a result of the expansion, was lower (ranging from 1.95 to 2.88 mm in the molar region and 2.18 to 2.66 mm in the premolar region) than the maxillary expansion (ranging from approximately 2.59 to 3.31 mm in the molar region and 3.48 to 4.40 mm in the premolar region), which in turn was low when compared to the interdental width of the anchor teeth (from approximately 6.11 to 6.70 mm in the molar region and 6.17 to 6.33 mm in the premolar region. On the basis of these results, an upwardly decreasing expansion gradient was observed, in agreement with the results of previous studies [2, 29] and with a previous systematic review on the topic, in which the MARPE reportedly provided a skeletal component of expansion ranging from 25 to 61% [28, 29, 35]. It is worth mentioning that the skeletal/dentoalveolar expansion pattern may be attributed to individual craniofacial characteristics, such as the degree of midpalatal suture maturation. There is a need to investigate the relationship between the craniofacial characteristics and the alveolar apparatus after use of the MARPE [36, 37]. In the axial plane, the increase in the NPF distance was greater than the increase in the GPF region, indicating a V-shaped expansion of the dental arch, decreasing from anterior to posterior (NPF > GPF). It might be attributed to the anatomic distance between the GPF and the point of expansion force application, as well as to the complex posterior articular surface with the cranial base which limited the effect of the expansion forces.

Carlson et al. [32] reported significant expansion in the zygoma area using MARPE. The relatively small average expansion in the zygoma area observed in the present study may be attributed to individual variations, usually observed in a prospective study design. Even when MARPE was applied, the line of action still passes below the presumed center of resistance of the maxilla, which is grasped by several bony structures on top, including the calvarial bones. Accordingly, a triangular expansion is a common finding regardless of the presence of miniscrews (Table 3) [2, 29]. Moreover, there was no significant difference between the RPE and MARPE groups with regard to expansion at the ZMS, indicating that the zygomatic expansion may be attributed to both the tooth-borne part and the miniscrews. This triangular expansion allows a greater enlargement of the dentoalveolar area, which enables a greater perimeter enlargement compared to the amount of basal bone expansion. Interestingly, all groups exhibited reduction in the basal bone dimension during the consolidation period (T2-T1), supporting the findings by Garib et al. [12] who demonstrated buccal bone dehiscence in younger patients. The significant difference between the M-NW in the RPE and MARPE groups at T1-T0 and T2-T0 indicates that the miniscrews may have played a role in the magnitude of skeletal expansion, and also, maintaining the maxillary bone segments during consolidation.

In our study, the MARPE and RPE groups showed similar dentoalveolar changes except for the maxillary width (PM-MW, M-MW). Intergroup differences in the maxillary width may be because these measurements are a part of the alveolar bone area. Buccal inclination of the anchor teeth resulting from maxillary expansion has been a common finding [9]. We also observed increases in anchor teeth inclinations in both the RPE and MARPE groups. In both type of interventions, the inclination of the first molars significantly increased after expansion (3.4°–3.94°) and decreased during consolidation, confirming the results of previous studies [9]. Therefore, the presence of miniscrews does not appear to guarantee the translation of the anchor teeth to the buccal side.

In both groups, buccal bone thickness of the first molars decreased by approximately 0.4–0.7 mm, whereas palatal bone thickness increased by 0.5–0.9 mm immediately after the RPE and MARPE expansion, indicating buccal displacement of the anchor teeth within the alveolar bone, in accordance with the results of previous studies [9, 12].

Garib et al. [12] reported that rapid maxillary expansion induced buccal bone dehiscences on the anchorage teeth, especially in participants with thinner buccal bone plates. In other studies, the main factors contributing to gingival recession were buccally located or displaced teeth, bone dehiscences, and a thin periodontal biotype [38–40]. According to the results of the present study, as the decrease of the anchorage teeth’s buccal bone thickness was minimized in the MARPE group, it may be assumed that the chances of bone dehiscences were also reduced.

However, regarding bone thickness changes around the premolar and the first molar, significant differences between RPE and MARPE group were observed in both buccal and palatal bone thicknesses, implying the more stable positioning of the anchor teeth in the MARPE group during the expansion and consolidation periods. Admitting the limitation of the study, it can be claimed that the miniscrews play a role in maintaining the anchor teeth within the maxillary basal bone segment during the consolidation period, enforcing periodontal safety during orthopedic expansion. The elasticity of the basal bone may produce a constant force from the zygomatic buttress against the anchor teeth. Therefore, without miniscrews, at the end of the consolidation period, the actual amount of basal bone may be decimated. According to our study, MARPE played a critical role by eliminating some negative side effects of the maxillary expansion procedure, whereas RPE resulted in buccal alveolar bone thinning of the anchor teeth. A significant positive correlation between the amount of expansion and periodontal variables of the anchor teeth during expansion (T1-T0) in the MARPE groups indicated the consistent relationship between the amount of expansion and the changes in the palatal bone plate thickness of premolar region. In contrast, the lack of correlation in the RPE groups appeared to imply the reduced predictability of the changes associated with the host factor (Table 6).

Taken together, within scope of the present study, it can be summarized that the reinforcement of RPE with miniscrews doesn’t affect the midpalatal suture separation ratio, however it appear to contribute to the maintenance of the basal bone during the consolidation period leading to less periodontal side effects, such as buccal dehiscence. Influence on the greater skeletal expansion was not evident. Future studies with long-term observations would be able to build the clinical significance of MARPE in terms of stability of transverse dimension and safety of the surrounding buccal bone.

## Conclusion

1. Midpalatal suture separation was observed in 90% (18/20) and 95% (19/20) of participants in the RPE and MARPE groups, respectively.
2. Both the RPE and MARPE groups exhibited significant triangular basal bone expansion (T1-T0) and skeletal relapse during consolidation (T2-T1).
3. A greater overall increase in the PM-NW, M-NW, and GPF was observed in the MARPE group during expansion and consolidation periods.
4. Under identical amounts of expansion, the MARPE group showed lower decrease in the BBPT in the premolar and molar regions after consolidation, indicating the miniscrew reinforcement may add a consolidation effect, maintaining the anchor teeth within the basal bone.

Taken together, the reinforcement of RPE with miniscrews appears to contribute to the maintenance of the basal bone during the consolidation period leading to less periodontal side effects, such as buccal dehiscence.

## Data Availability

All data generated or analyzed during this study are included in this published article.

## Abbreviations

RPE: Rapid palatal expansion
MARPE: Miniscrew-assisted rapid palatal expansion
CONSORT: Consolidated Standards of Reporting Trials (CONSORT)
CBCT: Cone beam computed tomography
FZS: Frontozygomatic suture
ZMS: Zygomaticomaxillary suture
NW: Nasal width
NPF: Nasopalatine foramen
GPF: Greater palatine foramen
MPS: Midpalatal suture
MW: Maxillary width
IDW: Interdental width
DI: Dental inclination
BBPT: Buccal bone plate thickness
PBPT: Palatal bone plate thickness
